# Linking Sport Participation With Prosocial and Antisocial Behaviours in Adolescents: The Moderating Role of Perceived Coach and Physical Education Teacher Character–Building Competency

**DOI:** 10.1002/ejsc.70122

**Published:** 2026-02-14

**Authors:** Nicholas Stanger, Susan H. Backhouse, Eoin Murray, Jim McKenna

**Affiliations:** ^1^ Carnegie School of Sport Leeds Beckett University Leeds UK; ^2^ University Academy 92 Manchester UK

**Keywords:** adolescence, antisocial behaviour, morality, prosocial behaviour, violence

## Abstract

Research investigating the relationship between sport participation and day‐to‐day antisocial and prosocial behaviours in adolescents has revealed mixed findings. However, research investigating whether social factors reflective of how sport is facilitated could moderate these relationships has received scant attention. This study examined whether perceived coach or physical education (PE) teacher character–building competency moderated relationships between sport participation and both day‐to‐day prosocial and antisocial behaviours in adolescents. In a sample of 456 adolescents, coach or PE teacher character–building competency moderated how sport participation was linked with prosocial behaviour and violence. Specifically, there was a significant positive relationship between sport participation and prosocial behaviour when coach or PE teacher character–building competency was perceived high, but this relationship was negated when coach or PE teacher character–building competency was lower. Moreover, when coach or PE teacher character–building competency was perceived low, sport participation was positively associated with violence, but this association was negated when such character‐building competency was perceived higher. However, this latter moderating effect became nonsignificant after controlling for gender and sport club involvement. Perceived coach or PE teacher character‐building competency was also positively associated with day‐to‐day prosocial behaviours and inversely associated with day‐to‐day antisocial behaviours in adolescents. These findings offer valuable insight into how sport participation may contribute to adolescents' day‐to‐day prosocial and antisocial behaviours, alongside underscoring the importance of fostering sport environments that actively support moral character development.

## Highlights

- Sport participation was associated with more frequent engagement in day‐to‐day prosocial behaviours in adolescents when coaches or physical education teachers were perceived high in character‐building competency (e.g., being perceived to promote and model good sportspersonship, fair play and respect).
- When coaches or physical education teachers were perceived high in character‐building competency, this was linked with adolescents' reporting engagement in fewer day‐to‐day antisocial behaviours (including violence).
- This research reinforces that sport participation alone does not automatically lead to the development of moral character in young people. Instead, it highlights the critical role of the social environment fostered through sport. Specifically, our findings offer new insights into the importance of coaches and physical education teachers being perceived as competent in promoting sportspersonship, respect and fair play. When these adults are seen as effective role models for character building, adolescents are more likely to exhibit prosocial behaviours and less likely to engage in antisocial behaviours through their involvement in sport.

## 1 Introduction

Sport is a popular leisure activity (e.g., Black et al. 2022) considered as a potential avenue to influence social behaviours in young people and often considered a vehicle for positive youth development by promoting outcomes such as moral character and self‐discipline (e.g., Eime et al. 2013; Pennington 2017). However, existing research presents mixed findings regarding to how sport participation is associated with prosocial and antisocial behaviours in young people (e.g., Duan et al. 2022; Graupensperger et al. 2021; Spruit et al. 2016). These inconsistent results suggest that sport participation alone does not inherently foster moral character or ethical conduct (e.g., Doty 2006). Instead, they highlight the need to better understand factors that could moderate these relationships, such as those reflective of the social environment facilitated through sport. This research aimed to address this socially significant issue.

From a normative ethical perspective, several philosophical theories have been proposed to explain the foundations that guide individuals' moral behaviour and their judgements about what is good or bad and right or wrong. These include deontological ethics (which emphasises adherence to social norms and ethics), utilitarian ethics (which focuses on the consequences of actions and their ethical implications) and virtue ethics (which stress the importance of moral character and virtues in guiding behaviour) (e.g., Chaddha and Agrawal 2023; Kagan 1992). Although these theories differ in emphasis on what guides moral judgements and behaviour, they collectively suggest that morality (when viewed through a normative ethical framework) reflects socially accepted norms and codes of conduct. This may involve acting out of duty, considering the impact of one's actions on others or embodying personal virtues (e.g., Gert and Gert 2025). For instance, behaviours such as stealing or physical violence are widely regarded as morally wrong due to their violation of social norms and potential adverse consequences for others. Despite the lack of a universally agreed definition of morality (Dahl 2023; Gert and Gert 2025), it is commonly conceptualised as encompassing ways of treating others that involve principles of human welfare, justice, and rights, and regulating actions that may cause harm to others (e.g., Dahl 2023; Nucci 2001).

Cognitive developmental theories of moral development (e.g., Kohlberg 1981; Haan 1983) have been widely applied to understand morality in young people. It has been argued that these approaches are grounded in the assumption that universal moral values exist, that cognitive processes such as moral reasoning based on these values can be learnt and that moral constructs (e.g., values, reasoning and behaviours) are measurable (e.g., Stoll and Beller 1998). Scholars have also emphasised that morality is ultimately expressed through action (e.g., Blasi 1980), and that behaviour is the cornerstone of morality (Shields and Bredemeier 1995). Rest (1984) similarly advocated for moral theories and research to focus on understanding behaviour (Rest 1984), given that actions are what directly affects others (Kavussanu 2008).

Bandura's (1991) social cognitive theory of moral thought and action offers a psychosocial lens for understanding moral action. According to this theory, moral action is primarily governed by internal moral standards and other self‐regulatory processes. These moral standards about what is right and wrong are shaped via socialisation, particularly through our interactions with others, including observing, modelling or learning from influential others such as parents, peers, teachers and sport coaches. Bandura (1999) further distinguished between two aspects of morality—proactive and inhibitive. Proactive morality reflects an individual's capacity to act humanely and engage in positive social behaviours, whereas inhibitive morality involves the ability to refrain from harmful or inhumane actions. Thus, higher levels of morality are demonstrated both through the enactment of positive social behaviours (i.e., prosocial behaviours) and the avoidance of negative social behaviours (i.e., antisocial behaviours) (Bandura 1999; Kavussanu 2008).

Prosocial behaviours are typically defined as voluntary actions intended to help or benefit others (Eisenberg and Fabes 1998). In adolescence, such behaviours may include helping, offering support or comforting others who are hurt, injured or in distress. In contrast, antisocial behaviours refer to actions that violate socially accepted norms and rules, often involving violence, vandalism, theft or other forms of harm (e.g., Simcha‐Fagan et al. 1986; van and Acker 2007). Among young people, antisocial behaviours can also manifest as defiance towards adult authority and problematic conduct in school. Adolescence is widely recognised as a critical developmental stage during which susceptibility to antisocial behaviour tends to increase. This vulnerability is strongly influenced by social contexts and relationships (e.g., Moffitt 1993).

### 1.1 Sport and Social Behaviour

Sport as a social context is widely considered a potential vehicle that could help to reduce adolescent antisocial conduct and potentially promote prosocial conduct. Social bonds’ theory has been applied to explain how sport could reduce antisocial behaviour in adolescents (Hirschi 1969). For instance, sport can build attachments with others (e.g., coaches and sport team members), distract attention from antisocial activities and strengthen societal beliefs and values (e.g., teach values such as fairness and discipline) (Hirschi 1969; Spruit et al. 2016). Scholars have also argued that sport can deter antisocial behaviours by socialising young people through exposure to conventional values (e.g., fairness and honesty), norms and rules and via developing psychosocial skills (e.g., self‐control) that can help regulate day‐to‐day behaviours (e.g., Arnold 1994; Eime et al. 2013).

On the other hand, sport could also promote antisocial behaviours. For instance, the competitive nature of sport may result in less mature moral reasoning whereby antisocial behaviours such as cheating or intentionally harming others are perceived as more legitimate, making them more likely (e.g., Bredemeier and Shields 1986). Research revealing that high school athletes reported lower mature moral reasoning than their nonathlete counterparts when presented with moral dilemmas offers some support for this proposition (e.g., Beller and Stoll 1995). If sport provides a context where antisocial behaviours are encouraged or legitimised, this could spill over to antisocial behaviours ‘off the field’ (e.g., Bloom and Smith 1996; Koss and Gaines 1993). Support for this potential spillover exists; for example, the culture of some sports around alcohol use (e.g., Kwan et al. 2014) has been linked with antisocial behaviours in day‐to‐day life (e.g., O’Brien et al. 2018).

In accordance with the contrasting propositions, research investigating the relationship between sport participation and antisocial behaviour has revealed mixed findings. A meta‐analysis of 51 studies revealed an overall null relationship (*r* = 0.01) between sport participation and delinquency (Spruit et al. 2016). However, this overall null relationship was an average effect carried by mixed and contrasting findings rather than a consistent lack of association. Indeed, studies contributing to the meta‐analysis identified all three possibilities—an inverse relationship (e.g., Eitle et al. 2021; Samek et al. 2015), a positive association (e.g., Begg et al. 1996; Endresen and Olweus 2005) or no association (e.g., McHale et al. 2005). Research examining the relationship between sporting participation and prosocial behaviour is also mixed, showing positive associations (e.g., Duan et al. 2022; Moeijes et al. 2018), very weak or null relationships (Graupensperger et al. 2021). These inconsistent findings suggest the likely presence of moderating factors.

In line with a cognitive moral developmental perspective (e.g., Doty 2006; Stoll and Beller 1998) and positive youth development in sport research (e.g., Holt et al. 2017), it has been argued that the social conditions of sport created by coaches and teachers—particularly those involving appropriate role modelling and supportive moral environmental conditions—are central to sport's potential to foster moral character and prosocial behaviour in young people. Through the process of socialisation, adolescents can learn and internalise values and moral standards that guide their behaviour (e.g., Bandura 1991). Consequently, the social environment within sport may influence how sport participation relates to everyday prosocial and antisocial behaviour in adolescents. Coaches and physical education (PE) teachers, as key figures in shaping young people's sporting experiences, are considered instrumental in cultivating environments that promote positive social behaviours (e.g., Côte and Gilbert 2009; Opstoel et al. 2020). However, research has yet to directly investigate how the social context promoted by these adults might moderate relationships between sport participation and day‐to‐day prosocial and antisocial behaviours. One potential moderating factor is the perceived character‐building competency of coaches and PE teachers.

### 1.2 Coach (or PE Teacher) Character–Building Competency

Coach character–building competency refers to the belief in the coaches' ability to influence athletes' personal development and positive attitude towards sport (Feltz et al. 1999). In the coaching efficacy conceptual model (Feltz et al. 1999), coaches high in character‐building competency would display more frequent moral character developmental behaviours. Although coach character–building competency may have some conceptual overlap with other constructs such as autonomy‐supportive coaching—which emphasises facilitating athletes' initiative taking, offering meaningful choices, acknowledging their perspectives and providing a rationale for requests (e.g., Mageau and Vallerand 2003)—it is distinguished by having a more direct focus on behaviours focused on positive moral development, such as facilitating good sportspersonship, fair play and respect for others. It is proposed when coaches display or perceived to display, such behaviours this could help teach prosocial behaviours in their players (e.g., Feltz et al. 1999). According to the coach effectiveness model (Horn 2002), sport participants' beliefs and attitudes are influenced (at least in part) by their perceptions, interpretations and evaluations of their coaches' behaviours, which are expected to have a role to play in the sport participants' behaviour. Empirical evidence supports this proposition. For instance, athletes' perceptions of their coach's character–building competency have been positively associated with prosocial behaviour and inversely associated with antisocial behaviour during sport competition (e.g., Boardley and Kavussanu 2009). However, despite these findings, it remains unclear whether these associations extend to adolescents' behaviour beyond the sport context.

There has been some debate regarding whether values such as fairness in sport are equivalent to fairness in nonsport contexts (see Pawlenka 2005), and also whether being taught values (or perceiving coaches being competent at teaching or promoting such values) in a sport context can be transferred to influence behaviour beyond the sporting environment. However, some scholars (e.g., Pawlenka 2005) have posited a *universalist* perspective, which argues that although examples of behaviours reflective of fairness in sport and nonsport contexts can be different, there is commonality in the general principles and concept of fair play (or fairness) in sport with fairness outside of sport. For example, fairness is reflective in norms and rules that facilitate equal opportunity of success and actions, which are performed within certain rules and principles of justice (e.g., Pawlenka 2005). Although the competitive nature of sport can foster egocentric reasoning, potentially increasing the likelihood of rule violations in pursuit of winning, if athletes internalise values such as fairness (or fair play) and respect within the competitive sport environment, these values may generalise to guide behaviour in noncompetitive everyday settings. Based on a *universalist* perspective, it is therefore plausible that in environments where coaches or PE teachers are perceived as competent in fostering moral character and promoting positive values, adolescents may be more likely to exhibit prosocial behaviours and less likely to engage in antisocial behaviours in their daily lives. Consequently, in sport settings where coach or PE teacher character–building competency is perceived to be high, one might expect stronger positive associations between sport participation and prosocial behaviour and stronger negative relationships between sport participation and antisocial behaviour. However, this proposition remains unexamined in empirical research.

### 1.3 Present Research

Existing research has produced mixed findings regarding the relationship between sport participation and behavioural outcomes, particularly in relation to antisocial behaviour (e.g., Spruit et al. 2016) and prosocial behaviour (e.g., Duan et al. 2022; Graupensperger et al. 2021). Despite growing interest in the developmental potential of sport, limited attention has been given to the social–contextual factors that could moderate these relationships. One such variable that warrants consideration is perceived coach or PE teacher character–building competency. Therefore, the present study aimed to examine whether sport participation is associated with adolescents’ day‐to‐day prosocial behaviour and antisocial behaviour, and whether coach (or PE teacher) character–building competency moderated any relationships. It was expected that coach or PE teacher character–building competency would accentuate any positive associations between sport participation and prosocial behaviour. It was also expected that coach or PE teacher character–building competency would attenuate any potentially positive associations between sport participation and antisocial behaviour (or strengthen any negative associations between sport participation and antisocial behaviour).

## 2 Methods

### 2.1 Participants

Participants were 456 adolescents (233 girls, 215 boys, and 8 nondisclosed) aged 11–16 years (*M* = 13.69; SD = 2.00) in the United Kingdom. Participants either took part in PE only (*n* = 116), engaged in extracurricular sport practice at school but not competitively (*n* = 45), represented their school (competitively) at sport but not a club (*n* = 20), competed for sports club but not the school (*n* = 93) or played/competed in sport for both their school and a club (*n* = 182). On average, participants reported engaging in a total of 1.70 (SD = 0.84) hours of organised (or compulsory) PE lessons per week and a total of 6.37 (SD = 5.01) hours in organised PE or sport overall.^1^

From the 295 participants who identified playing a sport for their school and/or at a club, the main sport that participants reported could be classified as either an individual (*n* = 120) or team (*n* = 161) sport (14 participants did not disclose), alternatively as either a collision (*n* = 164; e.g., basketball, hockey, martial arts, rugby and soccer) or noncollision (*n* = 117; e.g., badminton, cricket, golf, gymnastics, swimming and tennis) sport. A significant proportion of the participants also identified being involved in at least 2 (*n* = 207), 3 (*n* = 117) or 4 (*n* = 46) sports competitively.

To determine the minimum sample size to test for moderation in our study, we conducted a power calculation using G*Power (Faul et al. 2007). Specifically, to test for moderation for each behaviour (i.e., one dependent variable in each model and multiple predictors), we specified a small effect size (*f* ^2^ = 0.02) as this was the first study to test for such moderating effects, with conventional levels for power (1‐β = 0.80) and significance (*a* = 0.05), which yielded an estimated sample size of 395 participants.

### 2.2 Measures

#### 2.2.1 Sport Participation

To measure the extent of sporting participation, similar to previous research (e.g., Graupensperger et al. 2021), we conceptualised sport participation as the average time spent participating in sport each week. In this research, to calculate the average time spent participating in organised sport (including PE) in school and outside school for a club, participants were asked the following questions. How many hours a week do you spend in PE at school? How many hours a week outside of PE do you spend in practice/training in a sport (a) for the school? and (b) at a club? How many hours outside of PE do you spend competing in matches/games (a) for the school? and (b) at a club? A total number of hours was then calculated as a measure of sporting participation. Participants were also asked to identify their main sport if they played competitively for their school or a club. Then, participants were asked to list any other sports in which they participated competitively, alongside indicating whether they competed for the school, club or both, for each sport.

#### 2.2.2 Perceived Coach or PE Teacher Character–Building Competency

The 4‐item character‐building subscale from the Coaching Competence Scale (Myers et al. 2006) was used to measure perceptions about the character‐building competency of the PE teacher or the coach. Sport involvement typically varies among adolescents whereby some participants may only be involved in sport during physical education or school time, whereas others may also be involved in organised sport for clubs and teams outside of school (and potentially involved with several different sport clubs or teams). To help account for this, we asked participants to think about the sports coach or the PE teacher who they think plays the biggest role in their sporting experience and asked to complete each item in relation to the following statement ‘how good is your coach/PE teacher at…’. An example item is ‘teaching an attitude of fair play’. Participants responded to each item on a scale from 1 (*not at all good*) to 9 (*extremely good*). Myers et al. (2006) provided support for the validity and reliability of this subscale and has been used in research with adolescent youth sport (e.g., Malete et al. 2013) and high school student (e.g., Li et al. 2019) populations. For purposes of conciseness, although perceived coach character–building competency could refer to the coach or the PE teacher in the present research, we refer to this concept as coach character‐building competency through the remainder of the methods and results.

#### 2.2.3 Prosocial Behaviour

We used 10 items from the Prosocial Tendencies Measure—Revised (PTM—R; Carlo et al. 2003) to measure prosocial behaviour, which was developed and validated for young people aged 11–16 years. Specifically, we used the emotional (5 items), dire (3 items) and compliant (2 items) subscales. Each item was measured on a 5‐point scale anchored from 1 (*does not describe me at all*) to 5 (*describes me greatly*). Emotional prosocial behaviours refer to actions intended to benefit others in emotionally evocative situations (e.g., I usually help others when they are very upset). Dire prosocial behaviours refer to helping others under situations of crisis or emergency (e.g., I tend to help people who are hurt badly). Compliant prosocial behaviours refer to helping others when asked to (e.g., I never wait to help others when they ask for it). Carlo et al. (2003) provided psychometric support for these subscales. Similar with other research (e.g., Calderόn‐Tena et al. 2011; Carlo et al. 2018), we selected these three prosocial subscales as they were most closely interconnected and related to adaptive moral variables (e.g., empathy and internalised moral reasoning) in previous research (e.g., Carlo et al. 2003). Also, as these three subscales were highly and positively correlated during validation (*r*'s = 0.60–0.80; Carlo et al. 2003), and in the present study (*r*'s = 0.64 to 0.78), we used a composite score of these three subscales for prosocial behaviour aligned with previous research (e.g., Calderόn‐Tena et al. 2011; Carlo et al. 2018).

#### 2.2.4 Antisocial Behaviour

To measure participants' frequency of engaging in antisocial behaviour, we used the Bergen Questionnaire on Antisocial Behaviour (Bendixen and Olweus 1999). Specifically, we used three subscales as indicators of engagement in antisocial behaviours. We used 9 items from the *high prevalence* antisocial behaviour subscale, 4 items from the violence subscale, and the 3 items from the sanctions at school subscale. The antisocial behaviour subscale measures nonviolent behaviours such as problematic school behaviours (truancy, conflict with teachers and graffiti at school) and stealing or avoiding paying for things. The violence subscale measures physical forms of aggression (e.g., fighting) and public disruption, and the sanctions subscale measures frequency of being sanctioned by teachers for problematic behaviours. Although the scale was validated in young people aged 10–16 years, we slightly rephrased some items to improve readability and comprehension. In the antisocial behaviour subscale, we changed ‘had a violent quarrel with a teacher’ to ‘had a heated argument with a teacher’, ‘cursed at a teacher’ to ‘swore or cursed at a teacher’, and ‘Taken things worth less than NKr 200 from a store without paying’ to ‘taken things from a shop without paying’. For the violence subscale, we rephrased ‘got into a fight at a private party’ to ‘got into a fight at a party’.^2^ Other items used to measure violence were ‘got into a fight in a public place’, ‘beaten someone up so badly that they probably needed a doctor’ and ‘been noisy and rowdy in a public place’. An example item for the sanctions at school subscale is ‘been sent to the headteacher because of something you did’.

Participants responded to each item in relation to the following statement ‘In the last 6 months, have you….’, on a scale of ‘No’, ‘Once’, ‘Twice’ and ‘Three or more times’, which were scored as 0, 1, 2 and 3, respectively. A sum score was then taken for each subscale. Bendixen and Olweus (1999) provided psychometric support for the scale.

### 2.3 Procedure

Following ethical approval from the first author's institution, researchers contacted schools in the United Kingdom to facilitate access to participants. Following head teacher and parental consent, participants were invited to take part by a researcher/research assistant, which was typically during registration. Participants were informed verbally and via an information sheet about the purpose of the study, that participation was voluntary, responses were anonymous and stored confidentially, and about their right to withdraw, and they were invited to ask any questions about the research. Satisfied participants then completed a consent form and a questionnaire pack comprising the measures described above.^3^ After completion, participants sealed the questionnaire pack in an (blank) envelope and returned it directly to the researcher or research assistant. Participants were then thanked for participating and debriefed.

### 2.4 Data Analysis

Due to the antisocial behaviour, violence and sanctions at school scores being reflective of frequency or count data, square root transformation was performed on these variables (though means and standard deviations in the results’ table are raw scores for reporting purposes). To examine whether coach character–building competency moderated relationships between sport participation and each type of moral behaviour (prosocial behaviour and antisocial behaviours), we conducted moderation analyses aligned with Aiken and West (1991) using the PROCESS macro for SPSS (Hayes 2022). Specifically, as we were interested in one potential moderator, we applied Model 1 for regression analyses using the PROCESS macro. Moderation is supported when a significant interaction (i.e., when the confidence intervals do not cross zero) between mean centred moderator (i.e., coach character–building competency) and predictor (hours of sport participation) variables is revealed, when both the moderator and the predictor variable are included in the model. Any significant interactions were followed up by examining the strength of the relationships between the predictor (sport participation) and the outcome variable (i.e., different types of moral behaviours) at low (−1 SD), average (mean) and high (+1 SD) levels of the moderator (i.e., coach character–building competency).

## 3 Results

### 3.1 Preliminary Analyses

Data screening revealed 23 cases of missing data (0.36%) across the measures for coach character–building competency and prosocial behaviour. With such a low level of missing data, we assumed these were missing at random, so we calculated the mean of the remaining nonmissing items from the respective subscale (Tabachnick and Fidell 2013). Skewness and kurtosis values revealed no significant deviation from normality for any scale (univariate skewness < 1.5 and kurtosis < 2.2) (e.g., Kim 2013) and no extreme outliers. We also found no evidence of multicollinearity (i.e., tolerance ≥ 0.49, VIF ≤ 2.04). Descriptive statistics, scale internal consistencies and correlations are presented in Table 1.

**TABLE 1:** Correlations, descriptive statistics and internal consistencies.

	*M*	SD	*α*	*ω*	1	2	3	4	5	6
1. Coach CB competency (1–9)	5.92	2.01	0.92	0.92	—					
2. Prosocial behaviour (1–5)	3.75	0.76	0.92	0.92	0.16***	—				
3. Antisocial behaviour (0–27)	2.92	3.72	0.71	0.72	−0.21***	−0.13**	—			
4. Violence (0–12)	1.38	1.94	0.62	0.66	−0.08	−0.06	0.52***	—		
5. Sanction (0–9)	1.17	2.06	0.75	0.77	−0.08	−0.12**	0.59***	0.52***	—	
6. Sport participation	6.37	5.01	—	—	0.28***	0.09	−0.13**	0.02	−0.04	—
7. Gender	—	—	—	—	0.05	0.20***	−0.13**	−0.15***	−0.20***	0.02
8. Sport club involvement	—	—	—	—	0.28***	0.05	−0.10*	−0.01	−0.09	0.68***

*Note:* Sport participation refers to the hours of participation in sport and PE per week. Scale ranges for each measure are provided in parentheses. The *M* and SD for the raw sum scores of antisocial behaviour, violence and sanctions are reported in the table; however, the square root transformation values were used for correlational analyses. Gender was coded (0 = boys and 1 = girls), and the sample size for correlations involving gender was 448 due to 8 participants not disclosing their gender. Sport club involvement was grouped and coded as 0 = involved in PE and school sport only (i.e., no involvement in sport club) and 1 = involvement in sports club outside school. *α* = Cronbach's (1951) alpha coefficient; *ω* = McDonald's (1999) omega coefficient.

Abbreviation: Coach CB competency = Coach or PE teacher character–building competency.

^*^ *p* < 0.05.

^**^ *p* < 0.01.

^***^ *p* < 0.001.

### 3.2 Moderating Role of Coach Character–Building Competency

Table 2 shows the results from the moderated regression analyses in relation to whether coach character–building competency moderated the relationship between sport participation and each behaviour (i.e., prosocial behaviour, antisocial behaviour, violence and sanctions at school) without covariates and also with gender and sport club involvement included as covariates.

**TABLE 2:** Moderation analyses for sport participation and coach (or PE teacher) character building competency on behaviours.

	Prosocial behaviour			Antisocial behaviour (nonviolent)			Violence			Sanctions		
	*b*	SE	95% CI	*b*	SE	95% CI	*b*	SE	95% CI	*b*	SE	95% CI
Models with no covariates (*N* = 456)												
Sport participation	0.00	0.01	−0.01 to 0.02	−0.02	0.01	−0.04 to 0.00	0.01	0.01	−0.01 to 0.03	−0.00	0.01	−0.02 to 0.02
Coach CBC	0.06***	0.02	0.03 to 0.10	−0.11***	0.03	−0.16 to −0.05	−0.05*	0.02	−0.09 to −0.01	−0.04	0.02	−0.08 to 0.00
Sport part × coach CBC	0.01**	0.00	0.00 to 0.02	0.00	0.01	−0.01 to 0.01	−0.01*	0.00	−0.02 to −0.00	−0.00	0.00	−0.01 to 0.01
*F* (3,452)	6.26***			7.98***			2.63*			1.43		
*R* ^2^	0.04			0.05			0.02			0.01		
Interaction Δ*R* ^2^	0.02			0.00			0.01			0.00		
Models with covariates (gender and sport club involvement; *N* = 448)												
Gender	0.30***	0.07	0.13 to 0.42	−0.26**	0.10	−0.47 to −0.06	−0.25**	0.08	−0.41 to −0.09	−0.36***	0.08	−0.052 to −0.20
Sport club involvement	0.03	0.10	−0.17 to 0.23	−0.00	0.15	−0.29 to 0.29	−0.09	0.12	−0.32 to 0.13	−0.25*	0.11	−0.48 to −0.03
Sport participation	0.00	0.01	−0.02 to 0.02	−0.02	0.01	−0.05 to 0.01	0.02	0.01	−0.01 to 0.04	0.01	0.01	−0.01 to 0.04
Coach CBC	0.06**	0.02	0.02 to 0.10	−0.10***	0.03	−0.15 to −0.04	−0.04*	0.02	−0.09 to −0.00	−0.02	0.02	−0.07 to 0.02
Sport part × coach CBC	0.01*	0.00	0.00 to 0.02	0.01	0.01	−0.01 to 0.02	−0.01	0.00	−0.02 to 0.00	−0.00	0.00	−0.01 to 0.01
*F* (5,442)	6.55***			6.14***			3.29**			5.28***		
*R* ^2^	0.07			0.07			0.04			0.06		
Interaction Δ*R* ^2^	0.01			0.00			0.00			0.00		

*Note:* Unstandardised *b* coefficients are reported. Gender (0 = boys and 1 = girls) and sport club involvement (0 = no involvement in sport for a club and 1 = involvement in sport for a club) were included as covariates.

Abbreviations: Coach CBC = Coach or PE teacher character–building competency. Sport part × Coach CBC = product term formed via multiplying mean‐centred sport participation (in hours) and coach CBC. Interaction Δ*R* ^2^ = variance accounted for by the sport part × coach CBC product term in the respective model.

^*^ *p* < 0.05.

^**^ *p* < 0.01.

^***^ *p* < 0.0.

**FIGURE 1:**
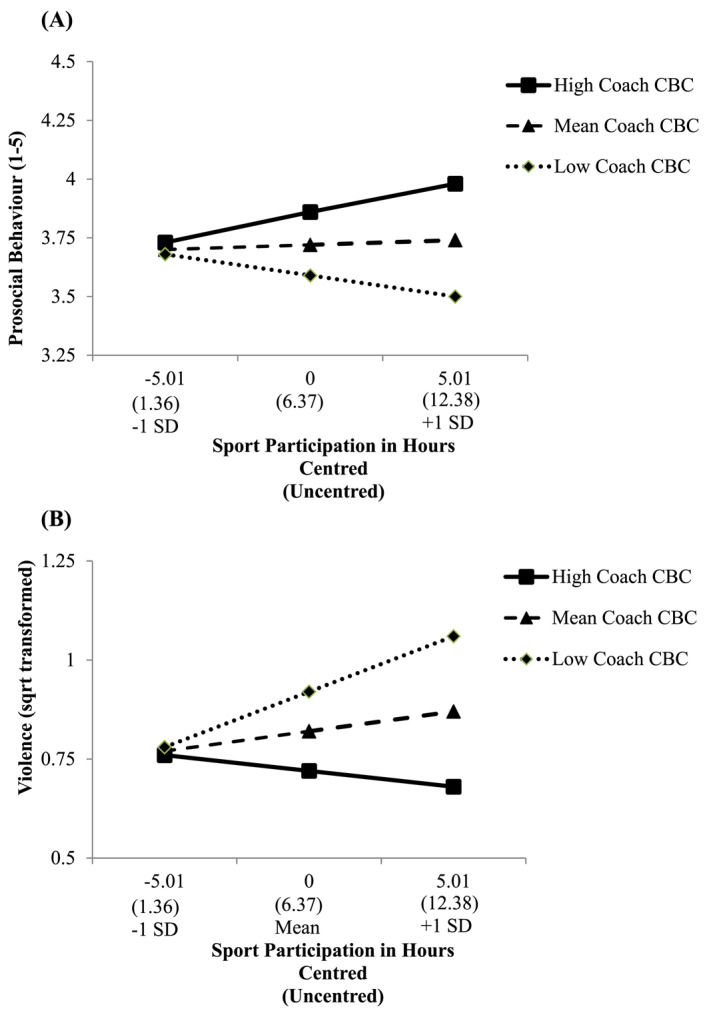
The moderating effect of coach or PE teacher character–building competency (Coach CBC) on the relationships between sport participation and prosocial behaviour (Panel A) and violence (Panel B).

In terms of prosocial behaviour, a main effect for coach character–building competency was noted reflecting that this was positively related to prosocial behaviour, but no main effect for sport participation was found. The sport participation × coach character–building competency interaction suggested that coach character–building competency moderated the relationship between sport participation and prosocial behaviour. As displayed in Figure 1A, sport participation was significantly and positively related to prosocial behaviour (*b* = 0.03, SE = 0.01, *t* = 2.60, *p* < 0.01 and 95% CI = 0.01 to 0.05) when coach character–building competency was high (+1SD; *M* = 7.93) but not when coach character–building competency was average (*M* = 5.92) (*b* = 0.00, SE = 0.01, *t* = 0.57, *p* = 0.57 and 95% CI = −0.01 to 0.02) or low (−1 SD; *M* = 3.91) (*b* = −0.02, SE = 0.01, *t* = −1.46, *p* = 0.14 and 95% CI = −0.04 to 0.01). This effect persisted after controlling for gender and sport club involvement.

For violence, coach character–building competency was a negative correlate, but sport participation was not related. However, a sport participation × coach character–building competency interaction was noted when no covariates were included in the model (*p* = 0.04). As displayed in Figure 1B, when coach character–building competency was low (−1SD; *M* = 3.91), sport participation was significantly and positively related with violence (*b* = 0.03, SE = 0.01, *t* = 2.12, *p = 0.03* and 95% CI = 0.00 to 0.05) but was not significantly associated when coach character–building competency was average (*M* = 5.92) (*b* = 0.01, SE = 0.01, *t* = 1.19, *p* = 0.23 and 95% CI = −0.01 to 0.03) or high (+1SD; *M* = 7.93) (*b* = −0.01, SE = 0.01, *t* = −0.73, *p* = 0.47 and 95% CI = −0.03 to 0.01). However, this interaction was negated when controlling for gender and sport club involvement (*p* = 0.14). Follow‐up analyses revealed that the effect was negated mainly due to including gender as a covariate. Specifically, when only sport club involvement was included as a covariate, the interaction remained significant (*b* = −0.01, SE = 0.00, *p* = 0.042 and 95% CI = −0.02 to −0.00). However, when only controlling for gender, the interaction became nonsignificant (*b* = −0.01, SE = 0.00, *p* = 0.17 and 95% CI = −0.02 to 0.00).

In terms of (nonviolent) antisocial behaviour, a main effect for coach character–building competency was revealed; coach character–building competency was a negative correlate of antisocial behaviour. However, no main effect for sport participation, nor sport participation × coach character–building competency interaction, was found. For sanctions at school, moderation analyses also revealed no main effects for sport participation or coach character–building competency, nor sport participation × coach character–building competency interaction.

## 4 Discussion

Although researchers have investigated how sport participation is associated with antisocial behaviour (e.g., Spruit et al. 2016) and prosocial behaviour (e.g., Graupensperger et al. 2021) in young people, the findings have been mixed, particularly in terms of antisocial behaviour. Further, limited attention has been paid to the social factors that may moderate these relationships. This research offers novel insights by examining the moderating role of coach (or PE teacher) character–building competency on these relationships. Although sport participation was not significantly associated with prosocial behaviour overall, perceived coach character–building competency of coaches and PE teachers moderated this relationship. Moreover, coach character–building competency was inversely associated with day‐to‐day antisocial behaviours and was positively associated with day‐to‐day prosocial behaviour, thereby extending previous research demonstrating these associations with sport‐specific prosocial and antisocial behaviours (e.g., Boardley and Kavussanu 2009). Taken together, these findings provide valuable new insights about how coaches and PE teachers could help facilitate sport as a context for regulating antisocial behaviours and enhancing prosocial behaviours in the day‐to‐day lives of sport‐involved adolescents.

Aligned with previous research reporting modest or negligible associations between sport participation and prosocial behaviour in adolescents (e.g., Duan et al. 2022; Graupensperger et al. 2021; Moeijes et al. 2018), the present study also found a small and nonsignificant association. However, this relationship was moderated by perceived coach (or PE teacher) character–building competency. Specifically, sport participation was positively linked to prosocial behaviour when coaches (or PE teachers) were perceived high in character–building competency, but this link was absent when such character‐building competency was perceived moderate or low. These findings suggest that when sport is delivered by coaches or PE teachers with a perceived emphasis on modelling good sportspersonship and instilling values such as fair play and respect, sport participation was linked to engagement in more frequent prosocial behaviour in adolescents' day‐to‐day lives. Thus, these findings support that when sport is delivered in a way that focuses on promoting moral character and associated values (e.g., fair play, good sportspersonship and respect), then this can help facilitate sport fostering positive psychosocial outcomes, including prosocial behaviour, in young people (e.g., Côte and Gilbert 2009; Opstoel et al. 2020; Stoll and Beller 1998).

Regarding antisocial behaviour, a small inverse association was found between sport participation and nonviolent antisocial behaviour, aligning with some previous research (see Spruit et al. 2016). However, sport participation was not associated with violence nor sanctions at school. Notably, coach or PE teacher character–building competency did not moderate the relationships between sport participation and (nonviolent) antisocial behaviours nor sanctions at school in the present study. A moderating effect for coach character–building competency on the link between sport participation and violence was noted, albeit this effect was attenuated after controlling for gender and competitive sport club involvement. Gender emerged as the main driver for this attenuation in the moderating effect, whereby similar to previous research (e.g., Baxendale et al. 2012), boys reported higher levels of violence than girls. Given that moderation effects are typically small, the moderating role of coach character–building competency on the link between sport participation and violence may not have been strong enough to contribute a significant unique variance beyond gender, in comparison to the stronger moderating role noted for the link between sport participation and prosocial behaviour. Nevertheless, the moderating effect was in the expected direction; sport participation was positively associated with violence when coach (or PE teacher) character–building competency was perceived as low but not when coach character building was perceived as high. These findings offer insights into the potential protective effects of the perceived coach (or PE teacher) character–building competency on violence in adolescents. They also suggest that coach (or PE teacher) character–building competency could potentially have a stronger moderating effect on the link between sport participation and more physically harmful forms of antisocial behaviour (e.g., violence) than with nonviolent forms of antisocial behaviour. Future research could further investigate this issue.

### 4.1 Limitations and Future Research Directions

Although the present study offers both novel and confirmatory findings, several limitations warrant consideration. This study was cross‐sectional, which can only infer relationships between variables, which restricts causal inferences regarding the direction of identified relationships. Future research employing longitudinal designs would be valuable in examining how sport participation and perceptions of perceived coach (and PE teacher) character–building competency over time, influences any changes in prosocial and antisocial behaviours. The present research focused exclusively on the perceived character‐building competency of the coach or PE teacher character building as a moderating variable. While this construct is theoretically and practically significant, other social factors may also moderate the relationship between sport participants and social behaviours, For instance, autonomy supportive or caring climates, which have been linked with prosocial and antisocial behaviour in adolescents in sport settings (e.g., Cheon et al. 2023; Gano‐Overway et al. 2009), may also play a moderating role in these relationships that warrant future investigation.

In the current research, we examined adolescents' perceptions of their coaches or PE teachers' character‐building competency who they perceived to play the biggest role in their sport involvement. This approach was designed to acknowledge the diverse nature of sport involvement during adolescence (e.g., Côte et al. 2007). However, it is important to acknowledge that other individuals (e.g., other coaches or PE teachers, parents and peers), could also contribute to the social environment of sport. Moreover, it is important to acknowledge that socialisation processes within sport are not always conscious or uniform and could be shaped by contrasting social experiences across different structural settings and sporting contexts, such as variations in coach or teacher personalities, behaviours and values that may not always be consciously recognised. Additionally, structural differences between school and club sport, such as the nature of competition or organisational culture, may further influence the socialisation process. Although sport club involvement was included as a covariate in the analyses to partially account for this,^4^ disentangling the unique contributions of each context was beyond the scope of this study. Notably, among the 295 participants involved in extracurricular sport, 182 participants reported involvement in both school and club sport, suggesting that many adolescents were simultaneously involved in multiple sporting environments, each comprising different coaches, teachers, peers and parental influences. To extend on the present findings, future research could aim to further disentangle the moderating role of coach character–building competency via investigating how these overlapping contexts, other social agents or relationships, and structural factors interact in shaping how sport is linked to day‐to‐day social behaviours in adolescents.

It is important to note that the sample in this study was drawn exclusively from the north of the United Kingdom, which may limit the generalisability of the findings to other regions or populations. Therefore, future research would benefit from testing the current findings across different geographical regions and cultural contexts to enhance generalisability. Although previous research has examined how sport types (e.g., team vs. individual sports and collision vs. noncollision sports) may be linked to sport participation and delinquency, any effects have been generally modest or nonsignificant (e.g., Spruit et al. 2016). Moreover, sport types do not appear to significantly influence the relationship between sport participation and prosocial behaviour (e.g., Moeijes et al. 2018). In the present study, many participants reported involvement in multiple sports spanning both team and individual sports, as well as collision and noncollision sports. This pattern of participation is consistent with the sampling phase of youth sport involvement (e.g., Côte et al. 2007). Therefore, given the range of sports that participants were involved in and the absence of data on the amount of time spent in each sport, it was not feasible to conduct sport‐type specific analyses in this study. Nonetheless, future research could consider address this limitation.

### 4.2 Practical Implications

The current findings also offer some applied implications in facilitating moral character development of young people through sport. Given that coach character–building competency facilitated sport being linked with more prosocial behaviours and was also inversely linked with antisocial behaviours in adolescents, sport coaches and PE teachers (and potentially others involved in the delivery of sport) would benefit from focusing on promoting and encouraging the importance of respect, fair play and sportspersonship. To aid this, coach and teacher education could consider approaches that enhance the effectiveness of promoting positive moral values in their practice. For example, activities where coaches and PE teachers are asked to consider positive moral values that they aim to develop in young people (e.g., fair play, honesty, respect) and how these values could be modelled and promoted in their practice may support the enhancement of perceived character‐building competency.

The findings of this study highlight the importance of coach and PE teacher education that includes structured training on how to explicitly teach moral values and facilitate their transfer beyond the sport context. One example of such an initiative is a multistage programme implemented in Singapore, designed for PE teachers and sport coaches, and led by an experienced educator with a background in teaching, coaching and coach education (Koh et al. 2016). The programme began with an introductory phase, during which PE teachers and coaches reflected on the importance of teaching values, identified key target values (e.g., respect) and were introduced to Kolb's (1984) experiential learning model. This framework was then applied to sporting scenarios to demonstrate how movement skills and moral values could be taught concurrently. Similar workshops have been used in other research to train sport coaches and PE teachers in values‐based education (e.g., Koh et al. 2017). The second phase involved planning, where coaches and PE teachers observed a demonstration on how to integrate movement skill instruction with teaching moral values during a soccer training session by the programme leader. Following this, they developed a 6‐week training plan applying these principles, with opportunities to receive feedback from the programme leader. The final practical phase required coaches and PE teachers to implement their teaching plan, during which they received constructive feedback (Koh et al. 2017). Additional resources, such as the *First Tee* golf‐oriented programme, have also demonstrated success in promoting respectful behaviour through strategies like coach modelling, role playing, peer modelling, instructing, use of coachable moments and reinforcement of rules and etiquette (Weiss et al. 2013). Such programmes and resources can enhance coaches' and PE teachers' perceived efficacy and competence in explicitly developing moral values and character development. They may also support reflective practice by encouraging effort, showing compassion when someone makes a mistake, helping others, and demonstrating respect to the opposition players, coaches, and parents (e.g., by shaking hands).

## 5 Conclusion

To conclude, sport participation alone does not inherently foster prosocial behaviour or mitigate antisocial behaviour in young people. The findings from the present study underscore the importance of how sport is delivered, particularly the role of coaches and PE teachers in shaping adolescents' moral conduct. Specifically, when coaches and PE teachers were perceived to possess higher character‐building competency, sport participation was positively associated with day‐to‐day prosocial behaviours. These results suggest that the intentional teaching of values such as fair play and respect, and the modelling of sportspersonship by youth sport coaches and PE teachers (and potentially others involved in delivering sport), may contribute to moral behaviours that extend beyond the sport context. In this way, sport can serve as a meaningful vehicle for promoting prosocial behaviour and discouraging antisocial behaviours among adolescents provided that sport is delivered and facilitated to foster such outcomes.

## Conflicts of Interest

The authors declare no conflicts of interest.

## Data Availability

As data sharing was not written into the approved ethics application for this study and we do not have consent from participants (nor head teachers or parents) to share individual scores, datasets comprising individual results cannot be made available. We only have consent to report results from analyses when combined across the whole sample.

## Endnotes

^1^
The original sample comprised 466 participants. Initial screening of the data revealed that 10 participants missed items on the antisocial behaviour and violence scale. As these scales were calculated as a sum score, these 10 participants were excluded from the final sample.

^2^
We did not include other antisocial behaviour subscales (e.g., illegal drugs, group/gang activity and low prevalence antisocial behaviour) and excluded one item from the high prevalence antisocial behaviour subscale (i.e., ‘Purposely destroyed or broken such things as windows, benches, telephone booths, or mailboxes’) for ethical reasons. This included a head teacher during initial stages of recruitment requesting this item from the high prevalence antisocial scale to be excluded due to perceived sensitivity of the item. As the omitted item was similar with an item in the low prevalence antisocial behaviour subscale about purposely destroying items, we believed the remaining items were sufficient in providing a measure of (nonviolent) *high prevalence* antisocial behaviour in the present research.

^3^
Prior to the main data collection, an initial sample of 17 participants (aged 11–12 years) completed the measures and were also asked to inform the researcher if they perceived there were any issues with the wording or their understanding of any items. No issues were reported per se, though a small minority checked for clarification with the researcher about the phrasing for two items from the high prevalence antisocial behaviour scale. Therefore, based on these interactions, we made very slight edits to the wording of these items to further enhance readability, which are incorporated above in the measures section (i.e., adapting ‘violent quarrel’ to ‘heated argument’ and ‘cursed’ to ‘swore or cursed’). No other issues were noted. Subsequent participants were also invited to inform the researcher or the research assistant if they required any further clarification of items (without overlooking any responses). No notable issues were reported.

^4^
Participants were also asked to indicate whether their (a) sports club coach/instructor or (b) PE teacher played the biggest role in their sporting experience. However, a total of 108 participants missed this question (likely due to presentation purposes whereby it was the only question not presented in a box or in a tabular format with coloured background, which may have meant some participants missed the question or did not realise they needed to specifically respond to this question). Therefore, we did not control for this in the main analyses due to the amount of missing data for this variable. From these responses, 202 participants reported the club coach/instructor, and 146 participants reported that their PE teacher played the biggest role in their sport experience. When conducting supplementary moderation analyses controlling for this variable with the available data (*N* = 348), the moderating effect for coach character–building competency on the link between sport participation and prosocial behaviour was still significant (*b* = 0.01, SE = 0.00, *p* = 0.01 and 95% CI = 0.00 to 0.02) but was not significant, albeit marginally, when also controlling for gender with this lower sample size (*b* = 0.01, SE = 0.00, *p* = 0.06 and 95% CI = −0.00 to 0.02). No significant moderating effect was noted for violence, sanctions or antisocial behaviour, when the reported biggest role in the sporting experience was controlled for in these supplementary analyses.
